# Feeder Cells Support the Culture of Induced Pluripotent Stem Cells Even after Chemical Fixation

**DOI:** 10.1371/journal.pone.0032707

**Published:** 2012-03-02

**Authors:** Xiao-Shan Yue, Masako Fujishiro, Chieko Nishioka, Takashi Arai, Eiki Takahashi, Jian-Sheng Gong, Toshihiro Akaike, Yoshihiro Ito

**Affiliations:** 1 Nano Medical Engineering Laboratory, RIKEN Advanced Science Institute, Wako-shi, Saitama, Japan; 2 Graduate School of Bioscience and Biotechnology, Tokyo Institute of Technology, Yokohama-shi, Kanagawa, Japan; 3 Support Unit for Animal Resources Development, Research Resources Center, RIKEN Brain Science Institute, Wako-shi, Saitama, Japan; Baylor College of Medicine, United States of America

## Abstract

Chemically fixed mouse embryonic fibroblasts (MEFs), instead of live feeder cells, were applied to the maintenance of mouse induced pluripotent stem (miPS) cells. Formaldehyde and glutaraldehyde were used for chemical fixation. The chemically fixed MEF feeders maintained the pluripotency of miPS cells, as well as their undifferentiated state. Furthermore, the chemically fixed MEF feeders were reused several times without affecting their functions. These results indicate that chemical fixation can be applied to modify biological feeders chemically, without losing their original functions. Chemically fixed MEF feeders will be applicable to other stem cell cultures as a reusable extracellular matrix candidate that can be preserved on a long-term basis.

## Introduction

Cultivation of pluripotent stem cells (PSCs) is inherently important for regenerative medicine. PSCs usually grow best when attached to other cells or to an extracellular matrix and grow traditionally in cultures with feeder layers (typically, mouse embryonic fibroblasts (MEFs) that have been irradiated or treated with mitomycin C (MMC) so that they can no longer divide. Treated MEFs with arrested cell cycles have been used to maintain embryonic stem (ES)/induced pluripotent stem (iPS) cells in an undifferentiated state, without losing their pluripotency [1]. The main reasons for this undifferentiation–maintenance effect of MMC-treated MEFs remain unclear. The most popular explanation is that MEFs secrete factors that are essential for maintaining PSCs in their undifferentiated state [2]. In addition, the importance of direct interactions between the microenvironment and PSCs is also pointed out [3]–[5].

Although MEFs are generally used in the culture of PSCs as feeder or nurse cells, the use of feeder cells is not desirable because of the laboriousness of the procedure (live cells must be prepared every time), the necessity for xeno-free culture for the cultivation of human ES cells, and the high risk of contamination during passages. The latter may happen when ES/iPS cells are digested from culture surfaces, as MEFs also detach from the surface, and contamination with MEFs may cause severe problems when ES/iPS cells are used for clinical purposes and may affect their differentiation efficiency. Therefore, many researchers are developing new culture systems to avoid using feeder cells.

To replace feeder cells, several external factors have been identified as important for the maintenance of the self-renewal ability of mES/iPS cells, including LIF [6], [7] and Wnt [8], [9]. Chemically defined media for PSCs have been reported by some researchers [10], [11]. Moreover, culture surfaces, including intelligent materials, immobilization of proteins, and synthetic polymer coating, have been also developed for PSCs [12]–[22]. However, these methods are still sometimes troublesome or have limitations, including the type of cell to which they can be applied and the cost.

In this research, we tried to use a more general and convenient method to produce a microenvironment for stem cell culture via a chemical modification of MEFs that renders them suitable for use as a reusable ECM that can be preserved on a long-term basis. To avoid the destruction of the proteins on the membrane of MEFs, we chose a low concentration of glutaraldehyde (GA) or formaldehyde (FA) as a fixation treatment. Chemical methods have been used to fix most of the proteins expressed in MEFs on a gelatinized surface, thus transforming the chemically fixed MEFs into feeders. Previously, Higashiyama et al. [23] used chemically fixed cells to investigate cell-membrane-associated growth factors, such as the heparin-binding epidermal growth factor (HB-EGF). These authors demonstrated that the use of chemical fixation methods allowed the fixation of HB-EGF to the cell membrane while keeping its activity to stimulate other cells. This report suggested that mild treatment with chemical fixatives allowed the preservation of the activity of cell membrane proteins. Subsequently, a preliminary study reported that chemically fixed nurse cells support the growth and maintenance of hematopoietic stem cells [24] and of ES cells [25]. However, the undifferentiated states of the cultured stem cells were not evaluated sufficiently.

Here, we fixed MEFs chemically using GA or FA, followed by a freeze-dry step, to immobilize proteins on the membrane of MEFs. The chemically fixed MEFs were applied as a chemically modified biological ECM and their ability to maintain mouse iPS (miPS) cells, which was generated by Yamanaka group [26], was analyzed. Since the cells were induced by transfecting four genes (Oct3/4, Sox2, Klf4, and c-Myc) into MEFs derived from transgenic mice containing the Nanog-GFP-IRES-Puro^r^ reporter construct, the GFP can be used as a reportor of Nanog expression, which indicates the undifferentiated state of miPS cells. In this research, the undifferentiated state of cultured miPS cells was confirmed by Nanog expression and AP staining; the pluripotency of miPS cells was confirmed by neural differentiation induction and teratoma formation in mice.

## Results and Discussion

### Treatment of MEFs with GA or FA

**Figure 1:**
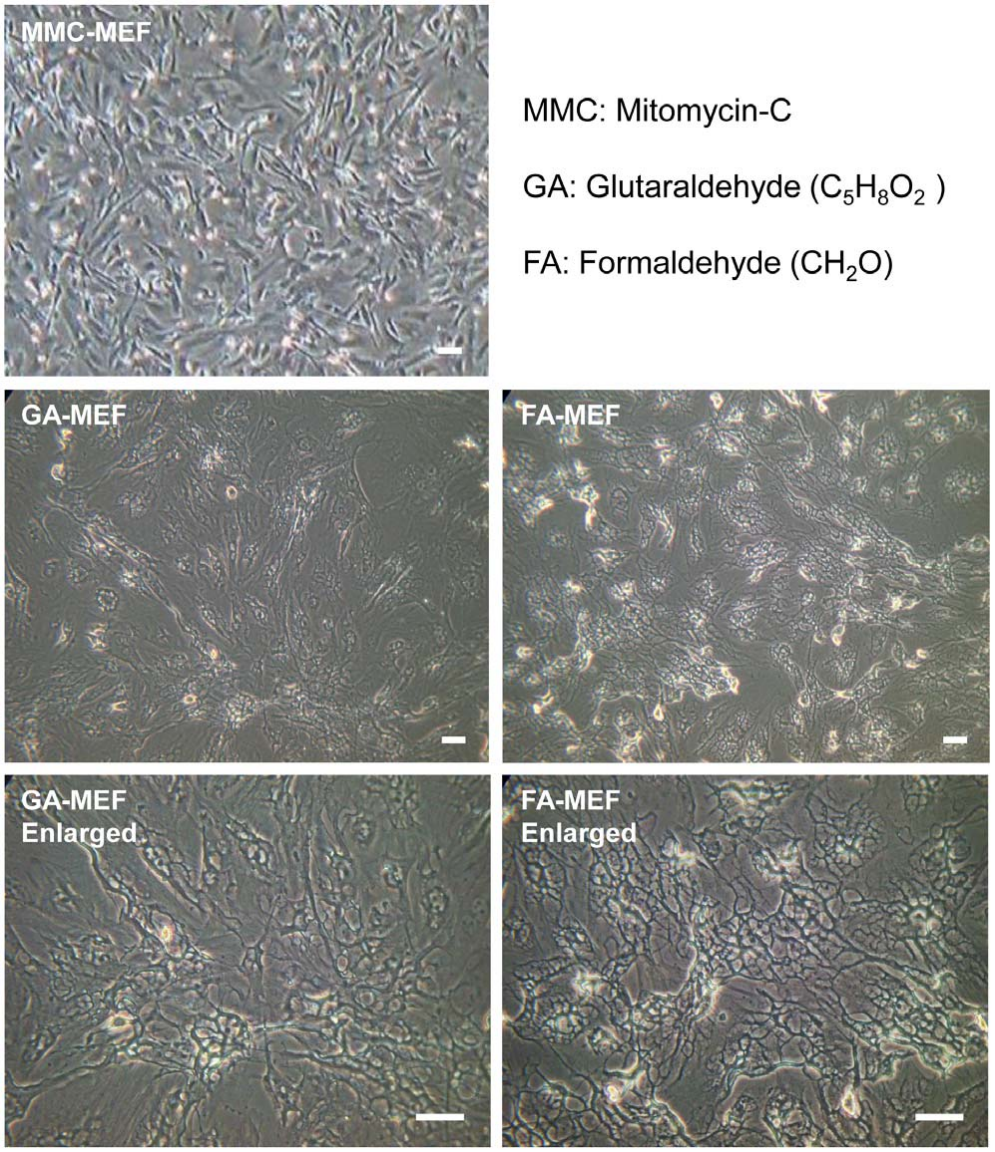
Morphology of MMC-treated MEFs and chemically fixed MEFs. MEFs were treated with 10 µg/mL MMC for 3–4 h, or fixed with 2.5% GA or 2.5% FA at room temperature for 30 min. After washing three times with PBS, fixed MEFs were freeze-dried and kept at room temperature. Scale bars, 50 µm.

GA and FA are two well-known chemicals that can be used in immunostaining or other experiments to fix cells while preserving the proteins. In this research, we tried to fix MEFs chemically via mild treatment with GA or FA, which leads to the immobilization of most of the proteins on the surface of MEFs or to their secretion from MEFs and absorption on MEF surfaces. The fixed MEFs exhibited a mesh-like morphology (Fig. 1) and the morphology was very similar after fixation with GA or FA. However, the morphology of fixed MEFs was different from that of MMC-treated MEFs.

### Morphology of miPS cells

**Figure 2:**
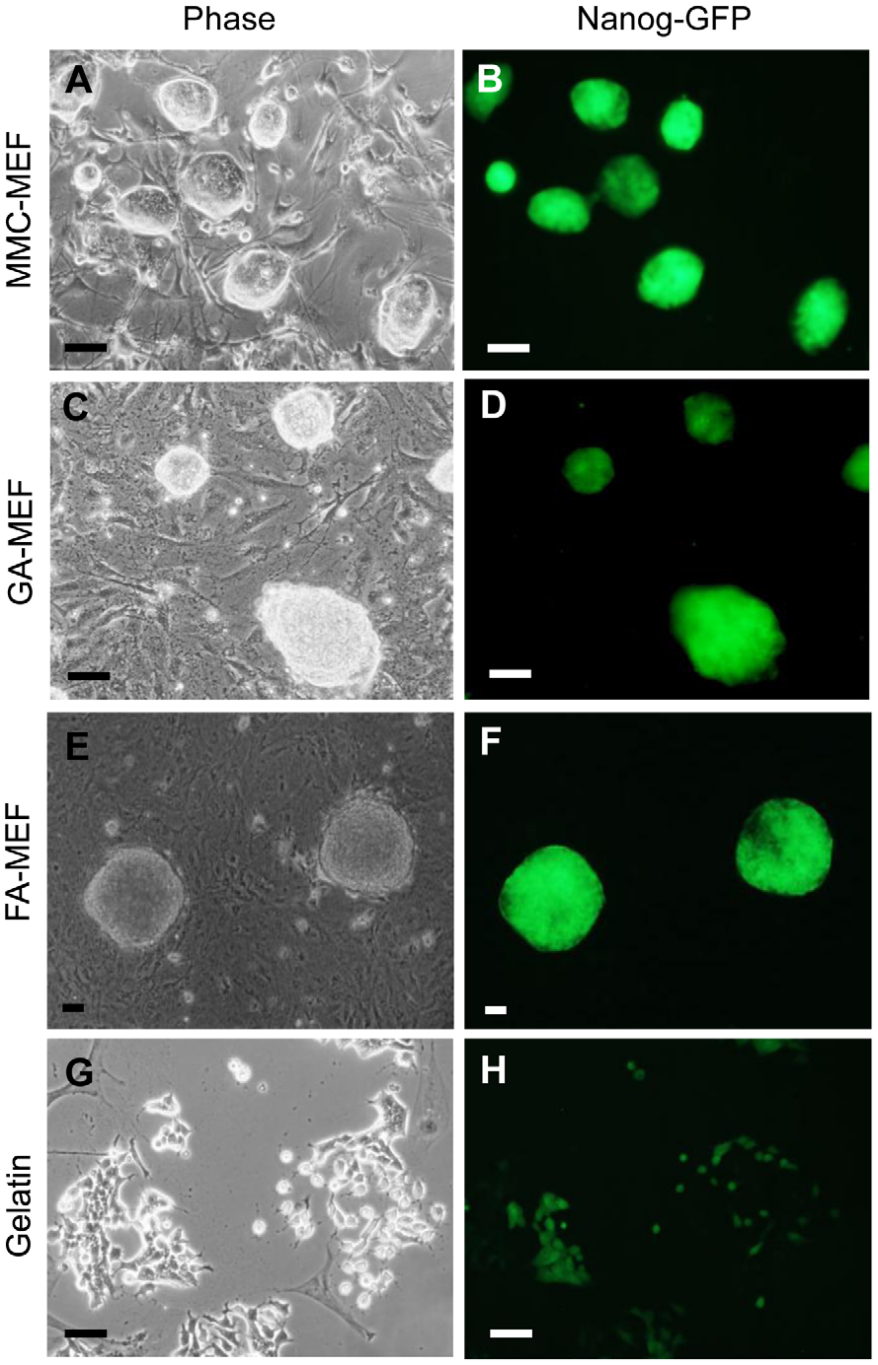
Morphology and Nanog-GFP expression in miPS cells cultured on MMC-treated MEFs, chemically fixed MEFs, or gelatin-coated surfaces. The green color indicates Nanog-GFP expression. Scale bar, 100 µm.

miPS cells formed colonies and maintained Nanog expression after six passages on GA-MEFs or FA-MEFs, which is very similar to what was observed for those cultured on MMC-treated MEF feeders (Fig. 2). Although the morphology of fixed MEFs was different from that of MMC-treated MEFs (Fig. 1), the morphology of miPS cells was very similar on either fixed or live MEFs. This result indicates that the chemical treatment did not damage significantly the biological components of the fixed cells. In contrast, on the gelatinized surface, cells became flat clusters and the expression of Nanog decreased obviously (Fig. 2).

### Undifferentiated state of miPS cells

**Figure 3:**
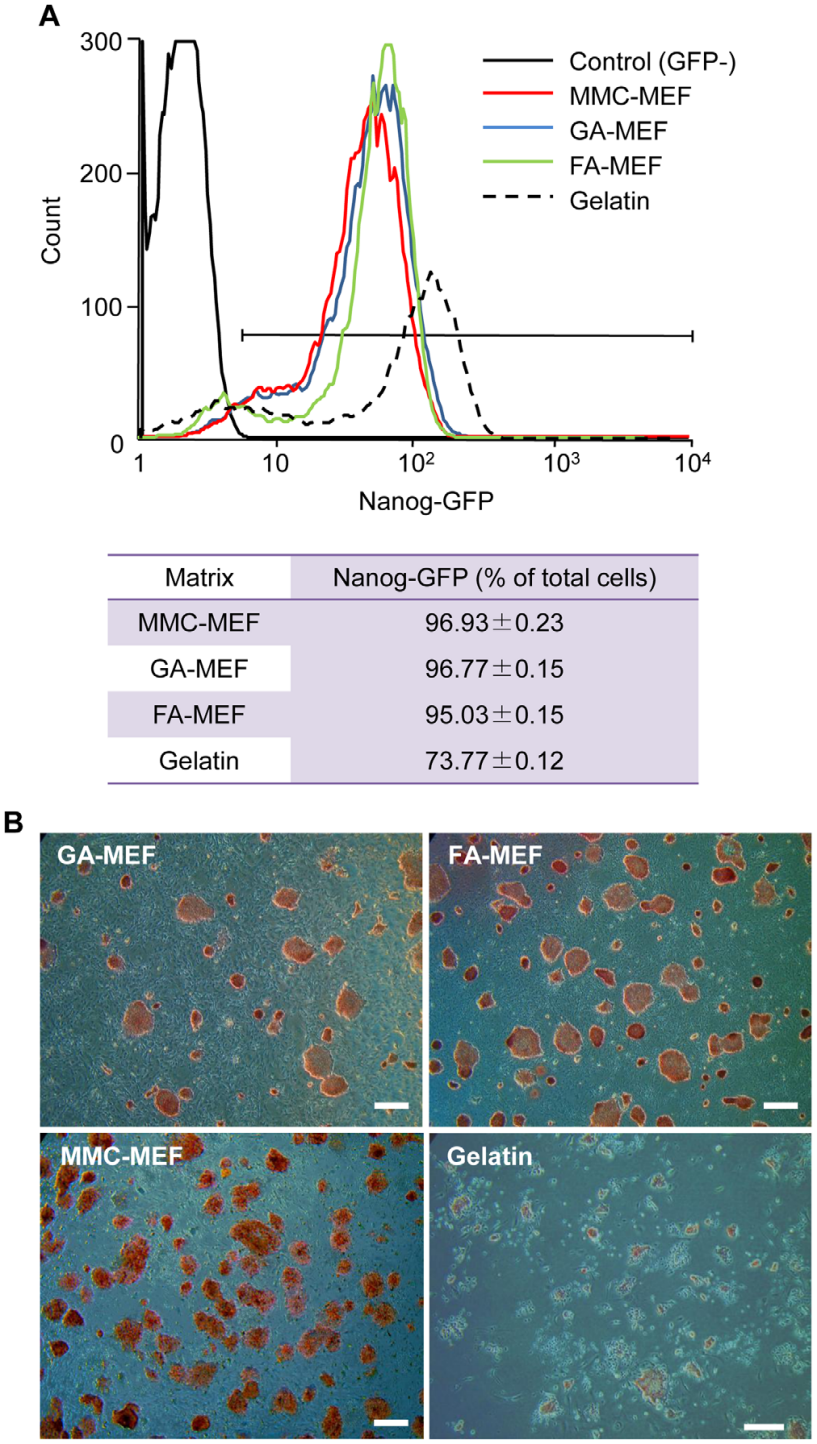
Maintenance of the undifferentiated state of miPS cells on MMC-treated MEFs or chemically fixed MEFs. (A) Percentage of Nanog-GFP-expressing miPS cells cultured on different matrices. A gelatin-coated surface was used as a control matrix, as it is not able to maintain miPS cells fully in an undifferentiated state. EB3 cells were used as a negative control, as they have no GFP fluorescence. The expression of Nanog was analyzed using a fluorescence-activated cell sorter (FACS) after 10 passages on MMC-MEFs, six passages on GA-MEFs or FA-MEFs, and one passage on a gelatinized surface. (B) Alkaline phosphatase (AP) activity of miPS cells cultured on chemically fixed MEFs. Histochemistry revealed AP activity (red) in miPS cells cultured on GA-MEFs or FA-MEFs after six passages. Scale bars, 500 µm.

Nanog was used as an indicator of the undifferentiated state of miPS cells. We used a FACS apparatus to analyze Nanog–GFP-expressing cells after six passages on GA-MEFs or FA-MEFs, to determine the percentage of undifferentiated cells cultured on chemically fixed MEF feeder cells. The results show that miPS cells cultured on GA-MEFs or FA-MEFs exhibited similar levels of expression of Nanog–GFP (Fig. 3A); more than 95% of cells expressed Nanog–GFP, which suggests that miPS cells maintain their undifferentiated state on chemically fixed MEFs. However, when cultured on a gelatinized surface, the percentage of Nanog–GFP-expressing cells decreased to about 74% (Fig. 3A).

AP activity is another indicator of the maintenance of an undifferentiated state. We confirmed AP activity in miPS cells cultured on GA-MEFs or FA-MEFs. The results show that miPS cells maintained their AP activity after six passages on chemically fixed MEF feeder cells (Fig. 3B). Together with the results of the FACS analysis, these findings indicate that the two types of chemically fixed MEF feeder cells can be used as substitutes for MMC-treated MEF feeder cells.

### Repeated use of chemically fixed MEFs

**Figure 4:**
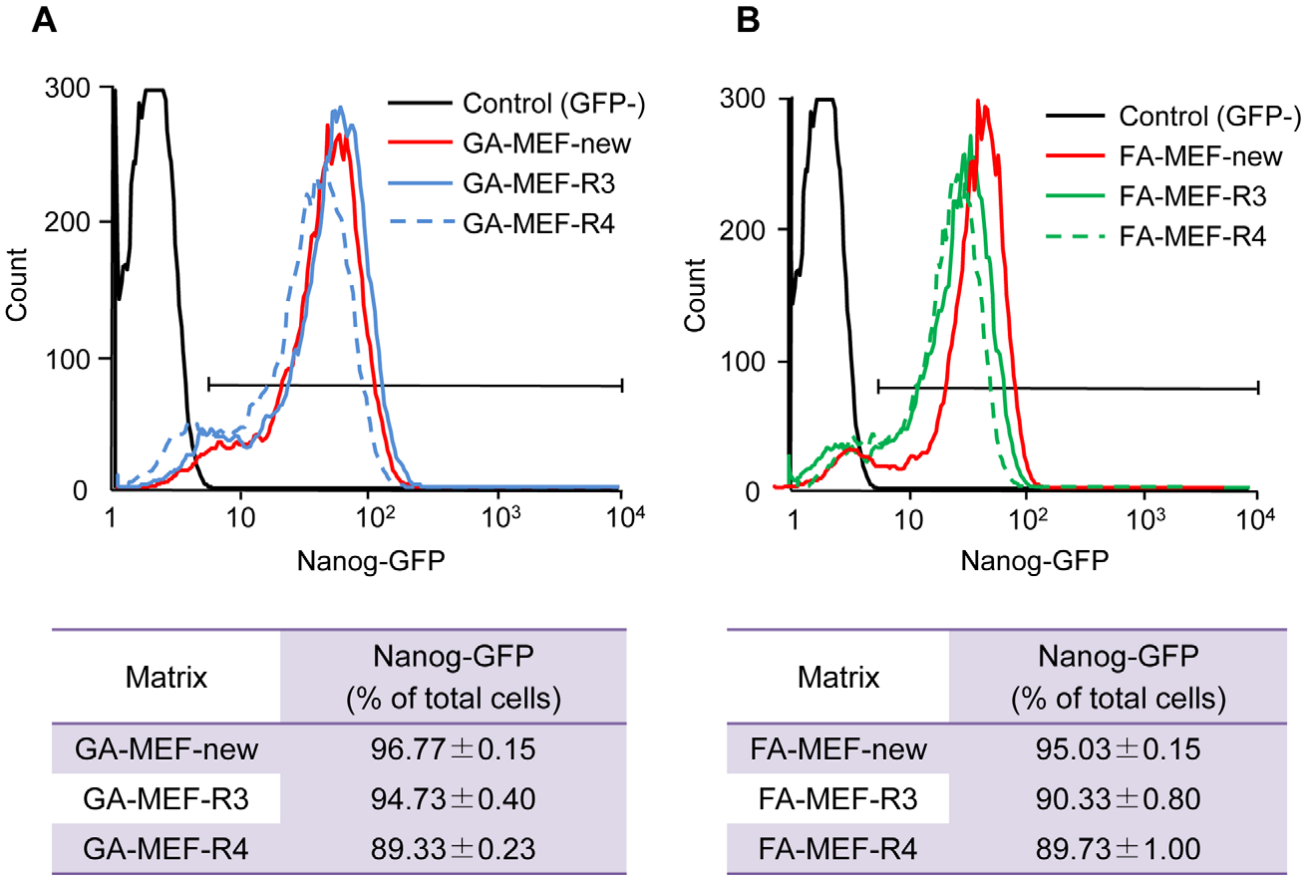
Percentage of Nanog-GFP-expressing miPS cells in reused chemically fixed MEFs. The expression of Nanog was analyzed by FACS after 10 passages on MMC-MEFs, six passages on new chemically fixed MEFs, or three passages on reused chemically fixed MEFs. (A) GA-fixed MEFs. (B) FA-fixed MEFs. MEF-new, chemically fixed MEFs used for cell culture for the first time; MEF-R3, chemically fixed MEFs reused for the third time; MEF-R4, chemically fixed MEFs reused for the fourth time.

During our experiment, we found that, after treatment with trypsin–EDTA for 5 min at 37°C and collection of miPS cells, chemically fixed MEF feeder cells remained immobilized on the surface of the dish. Therefore, we considered the possibility of reusing chemically fixed MEF feeder cells. We collected the dishes covered with fixed MEF feeder cells after removing miPS cells and washed them three times with PBS, to remove the remaining miPS cells completely. The dishes were used for further miPS cell culture. This treatment was repeated three (indicated as GA-MEF-R3 and FA-MEF-R3) or four (indicated as GA-MEF-R4 and FA-MEF-R4) times. miPS cells were seeded onto these reused feeders and passaged on these surfaces three times. Subsequently, cells were analyzed using FACS, to determine the percentage of Nanog–GFP-expressing cells (Fig. 4). The percentage of Nanog-expressing cells on GA-MEFs was about 95% and that on FA-MEFs about 90%. For maintaining the undifferentiated state of miPS cells, we consider that more than about 95% cells expressing Nanog demonstrate that we have achieved optimal conditions. According to this criterion, the GA-fixed MEFs were more suitable than FA-fixed ones. It is known that GA provides better fixation than FA by crosslinking reactions [27]. Similarly, GA is considered to fix the MEFs more firmly onto the dish surface, allowing them to be tolerant against trypsinization or PBS washing as used in this investigation.

### Differentiation of miPS cells into neural cells

**Figure 5:**
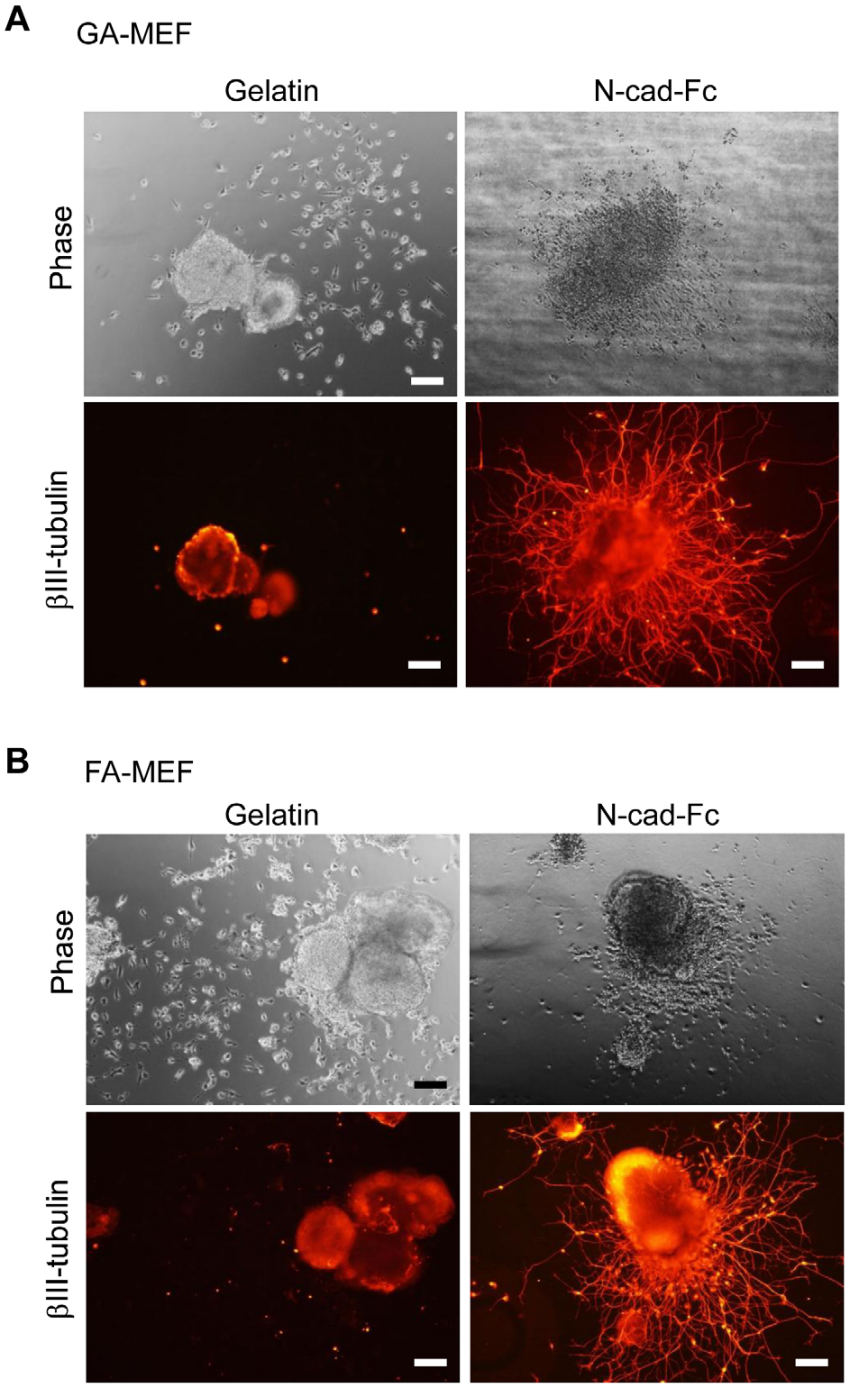
Neural differentiation of miPS cells cultured on FA-MEFs (A) or GA-MEFs (B). After six passages on FA-MEFs or GA-MEFs, miPS cells were collected and neural differentiation was induced using the SFEB method in KSR medium supplemented with 5 µM SB43152 n-hydrate and 5 µM CKI-7. After 5 days of suspension culture, cells were then transferred to different matrices. βIII-tubulin immunostaining was performed to detect the formation of neural cells. Scale bars, 200 µm.

We used the SFEB method to assess whether miPS cells maintained their differentiation ability when cultured on chemically fixed MEF feeder cells, as described in the “Materials and methods” section. The aggregates formed and transferred to N-cad-Fc-coated surfaces exhibited βIII-tubulin-positive neurite outgrowth (Fig. 5), indicating the maintenance of the ability to differentiate into neural cells. They exhibited a longer neurite stretch on N-cad-Fc-coated surfaces compared with gelatinized surfaces, which is in accordance with the results of our previous report [28].

### Pluripotency of miPS cells

**Figure 6:**
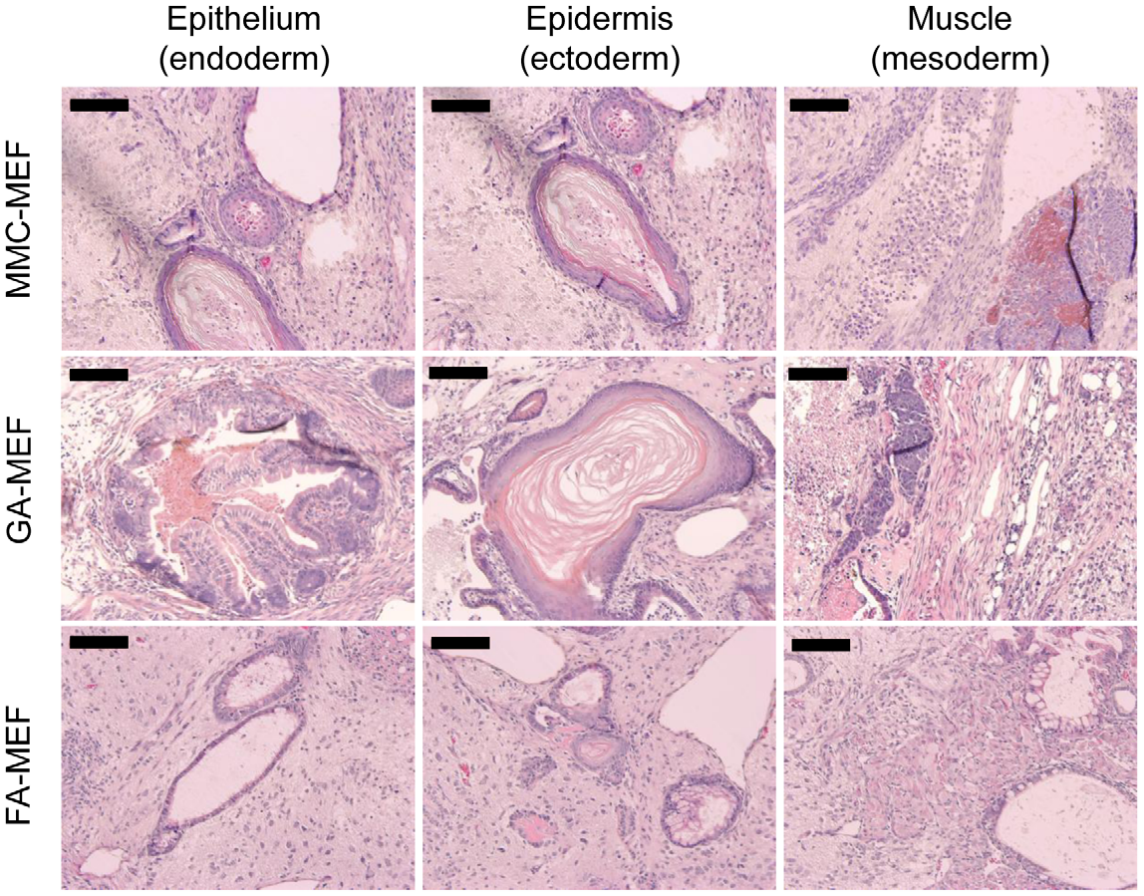
Teratoma formation from miPS cells cultured on GA-MEFs. After seven passages on GA-MEFs, miPS cells were collected and injected subcutaneously into the testis of 7-week-old BDF1 mice, to induce the formation of teratomas. Four weeks after injection, teratomas were fixed, sliced, and stained with HE. Scale bars, 100 µm.

The pluripotency of miPS cells cultured on GA-MEFs was confirmed further via the assessment of teratoma formation (Fig. 6). miPS cells were injected subcutaneously into the testis of 7-week-old BDF1 mice. Teratomas were isolated and fixed 4 weeks after transplantation. HE staining of paraffin sections demonstrated that all three germ layers were generated from miPS cells, including the ectoderm (indicated by epidermal and neural tissues), mesoderm (indicated by cartilage and muscles), and endoderm (indicated by epithelial tissues). These results suggest that miPS cells cultured on GA-MEFs maintain their pluripotency to differentiate into tissues of the three germ layers.

In this study, we developed two types of chemically fixed MEF feeders and applied them to the maintenance of miPS cells. We confirmed that these chemically fixed MEF feeders maintained miPS cells in an undifferentiated state and maintained the pluripotency of miPS cells. Furthermore, the chemically fixed MEF feeders could be reused several times without affecting their functions.

There are several advantages of chemically fixed MEFs as follows. i) Chemically fixed MEFs were lyophilized and stored. Thus, the fixed MEFs could be used for a cell culture substrate at any time. ii) The chemically fixed MEFs are not detached and thus do not contaminate PCS cells, unlike living feeder cells. In the case of living feeder cells, it is very difficult to avoid contamination. It is very important to provide high-quality purified cells for future manipulation. iii) When grown on chemically fixed MEFs, miPS cells can maintain their undifferentiated state in the normal medium used for such cells, which avoids the troublesome preparation of conditioned medium or the utilization of expensive chemically defined medium. Considering these advantages, the methodology is applicable for the large-scale production of PSC cells requiring feeder cells.

## Materials and Methods

### Preparation of mouse embryonic fibroblasts (MEFs), MEF feeder cells, GA-fixed MEFs (GA-MEFs), and FA-fixed MEFs (FA-MEFs)

All animal procedures were conducted according to the Guidelines for the Care and Use of Laboratory Animals of the RIKEN and were approved by the RIKEN Institutional Animal Care and Use Committee (Approval ID: No. H23-2-218). MEFs were prepared from 13.5-day-old fetuses. Embryos were separated from the placenta and surrounding membranes. The brain and dark-red organs were dissected and discarded. Body parts were dissected into small pieces and washed twice with PBS, to remove blood as much as possible. Subsequently, tissue clusters were treated with 5 mL of trypsin–EDTA and incubated with gentle shaking at 37°C for 30 min, to separate cells completely. After addition of 25 mL of fresh medium, cell solutions were filtered using a mesh, to deplete unseparated tissue clusters, and separated cells were then transferred into 50 mL tubes. Cell solutions were centrifuged to collect MEFs in the pellet.

Mitomycin-C (MMC)-treated MEF feeder cells were prepared by incubating MEFs with DMEM medium supplemented with 10% FBS and 10 µg/mL of MMC at 37°C for 3–4 h. The medium was then changed to DMEM for miPS cells. MMC-treated feeder cells were incubated overnight at 37°C before use.

To prepare chemically fixed MEFs, confluent MEFs were washed with PBS. Subsequently, cells were incubated in a solution of 2.5% GA or 2.5% FA at room temperature for 30 min and were washed three times with PBS. Chemically fixed MEFs were then freeze-dried and stored at room temperature for further experiments.

### Preparation of gelatinized or N-cad-Fc-coated surfaces

To prepare gelatinized surfaces, tissue culture dishes were treated with 0.1% (w/v) gelatin for 2 h at 37°C. To prepare N-cad-Fc-coated surfaces, purified N-cad-Fc protein was diluted into the indicated concentration and the diluted solution was added to untreated polystyrene plates. After 2 h of incubation at 37°C, the surfaces were washed with PBS and incubated with a 0.25% BSA/PBS solution at 37°C for 2 h, to inhibit the unspecific attachment of cells to the surface.

### Culture of miPS cells

miPS cells (purchased from the RIKEN Cell Bank) [26] were cultured in DMEM supplemented with 15% (v/v) FBS, 1 mM sodium pyruvate (Invitrogen, Carlsbad, CA), 2 mM l-glutamine (Invitrogen), 0.1 mM nonessential amino acids (Chemicon, Temecula, CA), 0.1 mM 2-mercaptoethanol (Sigma, Saint Louis, MI), and 1,000 U/mL of LIF (Chemicon). For passaging, cells were treated with 0.25% trypsin–EDTA for 5 min at 37°C, to obtain single cells, and seeded onto different matrices, as indicated.

### Alkaline phosphatase (AP) activity assay

ES cells were fixed with 3.8% formaldehyde at room temperature for 10 min and were then washed with PBS. Alkaline phosphatase activity was detected using the Vector Red Alkaline Phosphatase Substrate Kit I (Vector Laboratories, Burlingame, CA).

### Flow cytometry analysis

To determine the percentage of Nanog–GFP-expressing cells cultured on different matrices, cells were treated with trypsin–EDTA and analyzed on a fluorescence-activated cell sorter.

### Induction of differentiation

The SFEB method was used for the neural differentiation of miPS cells [29]. Cells were seeded at a density of 5×10^4^ cells/mL in untreated PS dishes containing KSR differentiation medium (95% GMEM (Invitrogen), 5% knockout serum replacement (KSR, Invitrogen), 2 mM glutamine, 1 mM sodium pyruvate, 0.1 mM nonessential amino acids, and 0.1 mM 2-mercaptoethanol) and cultured in suspension for 5 days. Five micromolar SB43152 n-hydrate (SB, Wako) and 5 µM CKI-7 (CK, Sigma) were added during the suspension-culture period, to enhance the efficiency of neural differentiation [30]. The aggregates were then transferred to gelatinized or N-cad-Fc-coated surfaces, as indicated, and cultured for an additional 3 days in KSR differentiation medium without the addition of SB or CK. The expression of βIII-tubulin was assessed and used as a marker of neural cells using the immunofluorescence staining method.

### Immunofluorescence staining

Cells were fixed with Mildform 20 N (8% formaldehyde, pH 7.0–7.5; Wako Pure Chemical Industries) for 30 min and permeabilized with 0.2% Triton X-100/PBS for 2 min. After blocking with 1% BSA solution for 30 min at room temperature, the samples were exposed to an anti-neuron-specific βIII-tubulin antibody (clone TuJ-1; R&D Systems Inc.), followed by incubation with a Cy3-conjugated secondary antibody (Jackson ImmunoResearch Laboratories, West Grove, PA).

### Teratoma formation

A cell suspension of 0.5×10^6^ cells/20 µL was injected subcutaneously into one side of the testis of 7-week-old BDF1 mice. Four weeks after injection, the teratomas formed were fixed with 4% paraformaldehyde using the perfusion fixation method and embedded in paraffin. Sections with a thickness of 10 µm were stained with hematoxylin and eosin (HE).
